# Circulating mitochondrial‐derived microproteins at rest and in response to an acute bout of endurance exercise in individuals with cerebral palsy

**DOI:** 10.1113/EP093298

**Published:** 2026-06-25

**Authors:** Oscar Horwath, Linnéa Corell, Junxiang Wan, Emma Hjalmarsson, Julia Starck, Stefan M. Reitzner, Jessica Norrbom, Rodrigo Fernandez‐Gonzalo, Ola Kvist, Pinchas Cohen, Ferdinand von Walden, Sebastian Edman

**Affiliations:** ^1^ Division of Pediatric Neurology, Department of Women's and Children's Health Karolinska Institute Stockholm Sweden; ^2^ Department of Physiology, Nutrition and Biomechanics The Swedish School of Sport and Health Sciences Stockholm Sweden; ^3^ The Leonard Davis School of Gerontology University of Southern California Los Angeles California USA; ^4^ Medical Unit Allied Health Professionals, Women's Health and Allied Health Professionals Theme Karolinska University Hospital Stockholm Sweden; ^5^ Molecular Exercise Physiology Group, Department of Physiology and Pharmacology Karolinska Institute Stockholm Sweden; ^6^ Division of Clinical Pediatrics, Department of Women's and Children's Health Karolinska Institute Stockholm Sweden; ^7^ Division of Clinical Physiology, Department of Laboratory Medicine Karolinska Institute Stockholm Sweden; ^8^ Unit of Clinical Physiology Karolinska University Hospital Huddinge Sweden; ^9^ Department of Radiology Columbia University Irvine Medical Center New York New York USA; ^10^ Molecular Muscle Physiology & Pathophysiology Group, Department of Physiology and Pharmacology Karolinska Institute Stockholm Sweden

**Keywords:** cerebral palsy, frame running, humanin, MOTS‐c, physical exercise, SHMOOSE

## Abstract

Regular exercise using assistive movement devices, such as running frames, has emerged as a promising strategy to improve cardiorespiratory fitness in individuals with cerebral palsy (CP). However, the molecular pathways underlying these adaptations remain poorly understood. Here, we examined a novel class of signalling molecules, mitochondrial‐derived microproteins (MDPs), and assessed whether individuals with CP exhibit altered circulating levels compared with typically developing (TD) individuals at rest and following an acute bout of endurance exercise. Three groups were included: TD adults (31 ± 6 years), TD adolescents (16 ± 1 years) and adults with CP (25 ± 6 years). Individuals with CP were classified as Gross Motor Function Classification System (GMFCS) levels II−IV and had at least 3 months of frame running experience. Habitual physical activity, ultrasound‐derived muscle thickness, and peak oxygen uptake were assessed. The exercise session consisted of 45 min of frame running for individuals with CP and conventional running for TD participants. Blood samples were obtained before and 1 h after exercise, and plasma MDP concentrations were measured using in‐house enzyme‐linked immunosorbent assay. Adults with CP had reduced muscle mass and maximal oxygen uptake compared to TD individuals. Despite this, they exhibited basal circulating levels of MDPs, including humanin, MOTS‐c and SHMOOSE, comparable to TD adults and adolescents, with no associations with CP subtype or motor impairment severity. Following exercise, circulating MDPs showed no or only modest changes across groups, with no differences between CP and TD individuals. Overall, these findings suggest preserved mitochondrial‐derived signalling via MDPs in individuals with CP.

## Abstract (highlights)

- **What is the central question of this study?**
  Do adults with cerebral palsy (CP) exhibit altered circulating levels of mitochondrial‐derived microproteins (MDPs) compared to typically developing (TD) adults and adolescents?
- **What is the main finding and its importance?**
  Adults with CP exhibit circulating levels of MDPs, including humanin, MOTS‐c and SHMOOSE, comparable to TD adults and adolescents, both at rest and after an acute bout of endurance exercise. Despite lower muscle mass, aerobic capacity and exercise performance, mitochondrial signalling via MDPs appears to be preserved in individuals with CP.

## 1 INTRODUCTION

Cerebral palsy (CP) is the most common cause of lifelong motor disability, affecting more than 17 million people worldwide (Graham et al., 2016). CP results from an insult to the developing brain, leading to the formation of non‐progressive lesions (Graham et al., 2016). Although the aetiology and clinical presentation of CP are highly heterogeneous, common features include altered muscle tone (e.g., hypertonus), reduced joint range of motion, muscle weakness and lower skeletal muscle mass (Bolsterlee et al., 2025; Graham et al., 2016; von Walden et al., 2017). These impairments often lead to lower levels of physical activity (Maher et al., 2007), which in turn contribute to diminished cardiorespiratory fitness, particularly among individuals with severe motor impairments (Verschuren & Takken, 2010). Consequently, individuals with CP exhibit an elevated risk of secondary health complications, including cardiovascular disease, compared to typically developing (TD) peers (Peterson et al., 2015; Ryan et al., 2019). Exercise therefore represents a promising strategy to improve muscular and cardiometabolic health in individuals with CP (Hjalmarsson et al., 2020). Partly to facilitate this, assistive movement devices, such as running frames, are increasingly used to enable individuals with movement disabilities to engage in structured physical activity (Hjalmarsson et al., 2020). While it is known that exercise using running frames leads to increased fitness and muscle growth (Hjalmarsson et al., 2020), the molecular pathways governing these adaptations in CP remain largely unexplored.

Emerging evidence indicates that mitochondrial dysfunction plays a key role in the pathophysiology of skeletal muscle in CP (Edman et al., 2025). Beyond their canonical role in ATP production, mitochondria serve as central regulators of cellular signalling, stress responses and metabolic homeostasis (Suomalainen & Nunnari, 2024). Recently, a novel class of signalling molecules known as mitochondrial‐derived microproteins (MDPs; previously known as mitochondrial‐derived peptides) have been identified as important modulators of these functions. These small bioactive proteins, encoded by short open‐reading frames (ORFs) within the mitochondrial DNA, have been shown to protect against cellular stress and metabolic dysfunction (Kumagai et al., 2023; Merry et al., 2020; Yen et al., 2013). Endogenous levels of certain MDPs are altered in metabolic diseases (Du et al., 2018; Merry et al., 2020; Zhou et al., 2024). For instance, diabetic patients exhibit reduced circulating levels of humanin (Ramanjaneya et al., 2019), whereas administration of a humanin analogue in animal models enhances insulin secretion and restores glucose homeostasis (Kuliawat et al., 2013). Intriguingly, these microproteins also respond dynamically to physiological stimuli, such as exercise and metabolic stress, suggesting a broader role in systemic adaptation (Gidlund et al., 2016; von Walden, Fernandez‐Gonzalo et al., 2021; Woodhead et al., 2020). However, it remains unclear whether individuals with CP display a similar response, as several factors that may regulate circulating MDP levels, such as physical activity (Ryan et al., 2015), muscle mass (von Walden et al., 2017) and skeletal muscle mitochondrial function (Dayanidhi et al., 2021; von Walden, Vechetti et al., 2021), are altered in this population.

Beyond humanin, other MDPs, including mitochondrial open‐reading frame of the 12S rRNA (MOTS‐c) and small human mitochondrial ORF over serine tRNA (SHMOOSE), have garnered attention as regulators of metabolism and potential biomarkers of disease (Merry et al., 2020; Yen et al., 2025). For instance, MOTS‐c has been shown to enhance insulin sensitivity, promote glucose uptake in skeletal muscle, and protect against diet‐induced obesity in preclinical models (Lee et al., 2015). Far less is known about SHMOOSE, though early evidence indicates it may serve as a circulating biomarker for age‐related neurobiological disorders (Miller et al., 2023).

Although MDPs have emerged as promising biomarkers and regulators of metabolic health, their circulating levels have never been investigated in individuals with CP, a population marked by distinct muscle and mitochondrial abnormalities (Dayanidhi et al., 2021; von Walden, Vechetti et al., 2021). Understanding MDP regulation in individuals with CP may provide new insight into systemic metabolic health and inform the development of targeted exercise‐based interventions. Accordingly, this study aimed to quantify circulating MDPs in individuals with CP and compare them with levels observed in TD peers.

## 2 METHODS

### 2.1 Ethical approval

This study received approval from the Swedish Ethical Review Authority (DNR 2021–05116 and 2022−0665202) and was performed in accordance with the principles outlined in the *Declaration of Helsinki*, except that it was not registered as a clinical trial. All participants were fully informed about the experimental procedures and associated risks with enrolment. Written informed consent was obtained from all participants, as well as from the caregivers of children under the age of 18.

### 2.2 Participants

Thirteen adults with CP and 30 TD individuals (adults *n* = 17, adolescents *n* = 13) were included in this study (Table 1). Adults with CP were eligible if they had a confirmed diagnosis of spastic, dyskinetic or ataxic CP, were between 18 and 40 years of age, were able to understand and follow verbal instructions, had Gross Motor Function Classification System (GMFCS) levels II−IV, owned a running frame, had at least 3 months of frame running experience, and were able to exercise continuously on a running frame for 45 min. The clinical and functional characteristics of the individuals with CP are described in detail in Table 2. TD participants were between 13 and 40 years of age and reported that they were healthy and not taking any medication. Exclusion criteria for both groups included soft tissue surgery within the previous 6 months, bone surgery within the previous 12 months, and the presence of other medical conditions. Participant characteristics in each of the three groups are presented in Table 1.

**TABLE 1:** Participant characteristics.

	Typically developed (TD adults)	Typically developed (TD adolescents)	Cerebral palsy (CP)
Group size	*n* = 17	*n* = 13	*n* = 13
Males/females	8/9	7/6	5/8
Age (years)	31.4 ± 6.2	14.8 ± 1.3^*^	25.2 ± 5.7^*,#^
Height (cm)	173.0 ± 11.6	166.5 ± 7.1	163.1 ± 11.3^*^
Females Males	165.1 ± 9.1 182.0 ± 6.2	167.7 ± 3.8 165.8 ± 9.4^*^	158.6 ± 3.8^#^ 170.3 ± 15.9
Body mass (kg)	72.7 ± 14.6	58.6 ± 11.6^*^	54.0 ± 11.3^*^
Females Males	62.7 ± 9.0 84.0 ± 11.1	56.2 ± 5.3 60.9 ± 15.3^*^	53.3 ± 9.8 55.2 ± 14.7^*^
BMI (kg/m^2^)	24.2 ± 3.3	21.0 ± 3.2^*^	20.2 ± 3.2^*^
Females Males	23.8 ± 3.8 25.4 ± 3.2	20.2 ± 2.0 21.9 ± 4.0	21.1 ± 3.5 18.7 ± 2.3^*^
${\dot{V}}_{O_{2}\mathrm{peak}}$ (ml/min/kg BW)	46.3 ± 7.1	52.1 ± 5.5	33.6 ± 8.8^*,#^
Females Males	43.6 ± 6.6 50.8 ± 5.8	48.3 ± 4.3 55.2 ± 4.3	28.8 ± 5.1^*,#^ 42.0 ± 7.9^#^
Muscle thickness RF (mm)	22.8 ± 3.1	23.1 ± 4.9	16.9 ± 2.8^*,#^
Females Males	20.9 ± 1.4 25.6 ± 2.9	22.1 ± 3.3 23.8 ± 5.9	15.5 ± 1.3^*,#^ 18.9 ± 3.3
SGPALS	2.9 ± 0.7	3.6 ± 0.5^*^	2.8 ± 0.9^#^

Data presented as means ± SD. Data analysed using a one‐way ANOVA and Tukey's multiple comparison test. *Significantly different from TD adults. ^#^Significantly different from TD adolescents. Abbreviations: BMI, body mass index; CP, cerebral palsy; RF, rectus femoris; SGPALS, Saltin–Grimby Physical Activity Scale (1: physically inactive, 2: some light physical activity, 3: regular physical activity and training, 4: regular hard physical training for competition sports); TD, typically developed.

**TABLE 2:** Clinical and functional characteristics of participants with CP.

ID	Age (years)	Cerebral palsy	GMFCS	FR experience (years)
1	19	Dyskinetic	4	11
2	19	Dyskinetic	4	6
3	21	Spastic	4	12
4	21	Dyskinetic	2	N/A
5	22	Spastic	3	12
6	22	Dyskinetic	4	9
7	24	Dyskinetic	3	9.5
8	25	Ataxic	4	8
9	27	Spastic	3	5
10	27	Dyskinetic	3	10
11	30	Spastic	2	8
12	33	Spastic	4	7
13	38	Dyskinetic	4	5

Abbreviations: FR, frame running; GMFCS, Gross Motor Function Classification System.

### 2.3 Recruitment

Participant recruitment was conducted across Sweden over a 6‐month period in 2022, with the goal of including as many individuals as possible. Recruitment efforts involved contacting frame running clubs, attending frame running training sessions and promoting the study via social media. Information and a video about the study were made available on the website of the National Sports Confederation's Laboratory (Bosön, Stockholm, Sweden), which served as the venue for the acute experimental part of the study.

### 2.4 Anthropometry and physical activity levels

Body weight in individuals with CP was measured using a large electronic wheelchair‐accessible scale (Detecto 6550, Webb City, MO, USA). Height was measured using a tape measure, and participants were positioned either lying on an examination table or standing, depending on their functional ability. In cases where contractures limited full extension, segmental lengths between anatomical landmarks were measured and added together to estimate height. The same methods were applied when measuring weight and height in the TD participants. Additionally, all participants assessed their habitual physical activity level using the Saltin–Grimby Physical Activity Level Scale (SGPALS) (Saltin & Grimby, 1968). The levels are graded as follows: 1: physically inactive; 2: some light physical activity; 3: regular physical activity and training; and 4: regular hard physical training for competitive sports.

### 2.5 Ultrasound measurement for skeletal muscle thickness

Muscle thickness of the rectus femoris was assessed using B‐mode ultrasound (EPIQ 7, Philips Healthcare, Bothell, WA, USA) equipped with an eL18‐4 PureWave linear array transducer. Imaging was performed with a centre frequency of 18 MHz and a frame rate of 48 Hz to ensure high‐resolution capture of the musculoskeletal structures. To ensure that the muscle appearance was true to size and not influenced by external forces, all images were captured with minimal contact pressure, maintaining a thick layer of ultrasound coupling gel between the transducer and the skin surface. Measurements were obtained bilaterally in all participants at the mid‐thigh site, and statistical analyses were performed for both legs. As the results did not differ between sides, only the data from the right leg are presented. Several transverse images were captured to ensure consistency in measurements.

### 2.6 Cardiopulmonary characterisation

At a separate session, peak oxygen uptake (${\dot{V}}_{O_{2}\mathrm{peak}}$) was determined using an incremental exercise test to volitional exhaustion. TD participants completed the test on a standard treadmill (Rodby RL 2000E, Rodby Innovation AB, Vänge, Sweden), while individuals with CP used a running frame placed on a wide treadmill (Rodby v2, Rodby Innovation). Continuous breath‐by‐breath gas exchange was measured throughout the test using a metabolic cart (COSMED Quark Cardio Pulmonary Exercise Testing, Rome, Italy). Protocols for assessment of ${\dot{V}}_{O_{2}\mathrm{peak}}$ have recently been described in detail elsewhere (Corell et al., 2026).

### 2.7 The acute exercise bout

**FIGURE 1:**
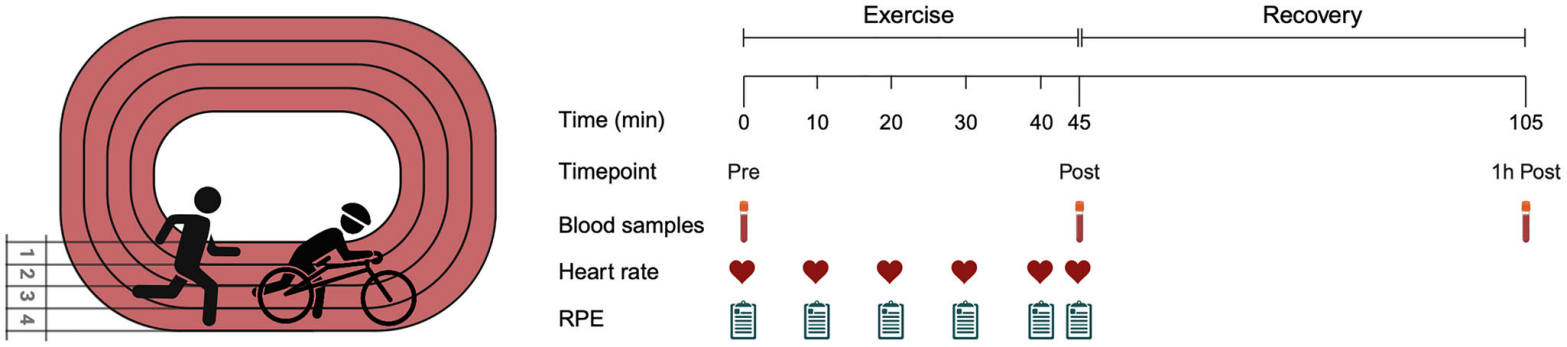
Schematic presentation of study design. RPE, rating of perceived effort.

Participants completed an acute 45‐min endurance exercise session performed on a 200‐m indoor track (for a detailed description of the protocol, see Kruse et al., 2024). This session consisted of frame running for individuals with CP and conventional running for the TD group. Participants were instructed to cover the maximum possible distance during the designated time and to sustain a Borg rating of perceived exertion (RPE) of 15 or higher throughout the session. Heart rate (HR), distance, and Borg RPE were continuously monitored. Average relative HR was calculated using 220 − age as the maximum value, unless a higher maximum HR was recorded during the 45‐min run or the ${\dot{V}}_{O_{2}\mathrm{peak}}$ test. A schematic overview of the experimental setup and timeline is shown in Figure 1.

### 2.8 Blood sampling

Venous blood was taken from a vein in the cubital fossa using a peripheral venous catheter. To reduce discomfort and pain, a local anaesthetic cream (EMLA, Aspen Nordic, Ballerup, Denmark) was placed on the skin at least 1 h before the first blood sample collection in adolescents and individuals with CP. Blood samples were collected at three time points: before (Pre), immediately after (post), and 1 h after (1 h post) the exercise session, as shown in Figure 1. The blood sample collected immediately after the exercise session (post) was used exclusively to measure lactate levels, and MDPs were not assessed at this time point. Blood samples were collected in plasma vacutainer tubes (BD, Franklin Lakes, NJ, USA). Plasma was prepared according to the manufacturer's recommendations, aliquoted, and stored at −80°C until further analysis.

### 2.9 Analyses of blood lactate and MDPs

Lactate levels were measured using a blood gas analyser (ABL800 Flex®, Radiometer Copenhagen, Denmark) in conjunction with the exercise session. Circulating levels of humanin, MOTS‐c and SHMOOSE in plasma were measured using an in‐house enzyme‐linked immunosorbent assay, as previously described (Chin et al., 2013; Lee et al., 2015; Miller et al., 2023). Post‐exercise MDP measurements were not available for all participants due to limited plasma sample availability, sampling error and one case of measurement failure. The number of samples included in each analysis is provided in the corresponding figure legends.

### 2.10 Statistics

Comparisons of basal humanin, MOTS‐c and SHMOOSE levels in TD adults, adolescents, and CP adults were performed using a one‐way ANOVA. A mixed‐effects model was also conducted on the pre‐ and post‐exercise values of humanin, MOTS‐c and SHMOOSE of all groups to assess the effect of exercise in each group individually. Correlations were calculated using Pearson's *r*, except for non‐continuous data (Borg RPE scale, SGPALS and relative HR_max_), in which case Spearman's rank correlation coefficient was used. Correlation coefficients were interpreted using conventional thresholds, with values of 0.10–0.29 considered low, 0.30–0.49 moderate, and ≥0.50 high. Statistical analyses were conducted using GraphPad Prism (version 10.1.2 for Windows; GraphPad Software, Boston, MA, USA). Data are presented as means ± SD.

## 3 RESULTS

### 3.1 Physiological characteristics

Three groups of individuals were included in the study: TD adults (aged 31.4 ± 6.2 years), TD adolescents (aged 14.8 ± 1.3 years) and adults with CP (aged 25.2 ± 5.7 years). The adults with CP were shorter (163.1 ± 11.3 vs. 173.0 ± 11.6 cm, *P *< 0.05), lighter (54.0 ± 11.3 vs. 72.7 ± 14.6 kg, *P *< 0.05) and had a lower BMI (20.2 ± 3.2 vs. 24.2 ± 3.3 kg/m^2^, *P *< 0.05) than TD adults. In addition, adults with CP generally had lower ${\dot{V}}_{O_{2}\mathrm{peak}}$ and muscle thickness compared to both TD adults and adolescents (*P* < 0.05; see Table 1). The TD adolescents reported higher average daily physical activity than both TD adults and CP adults (*P *< 0.05; see Table 1).

### 3.2 Plasma levels of MDPs at rest

**FIGURE 2:**
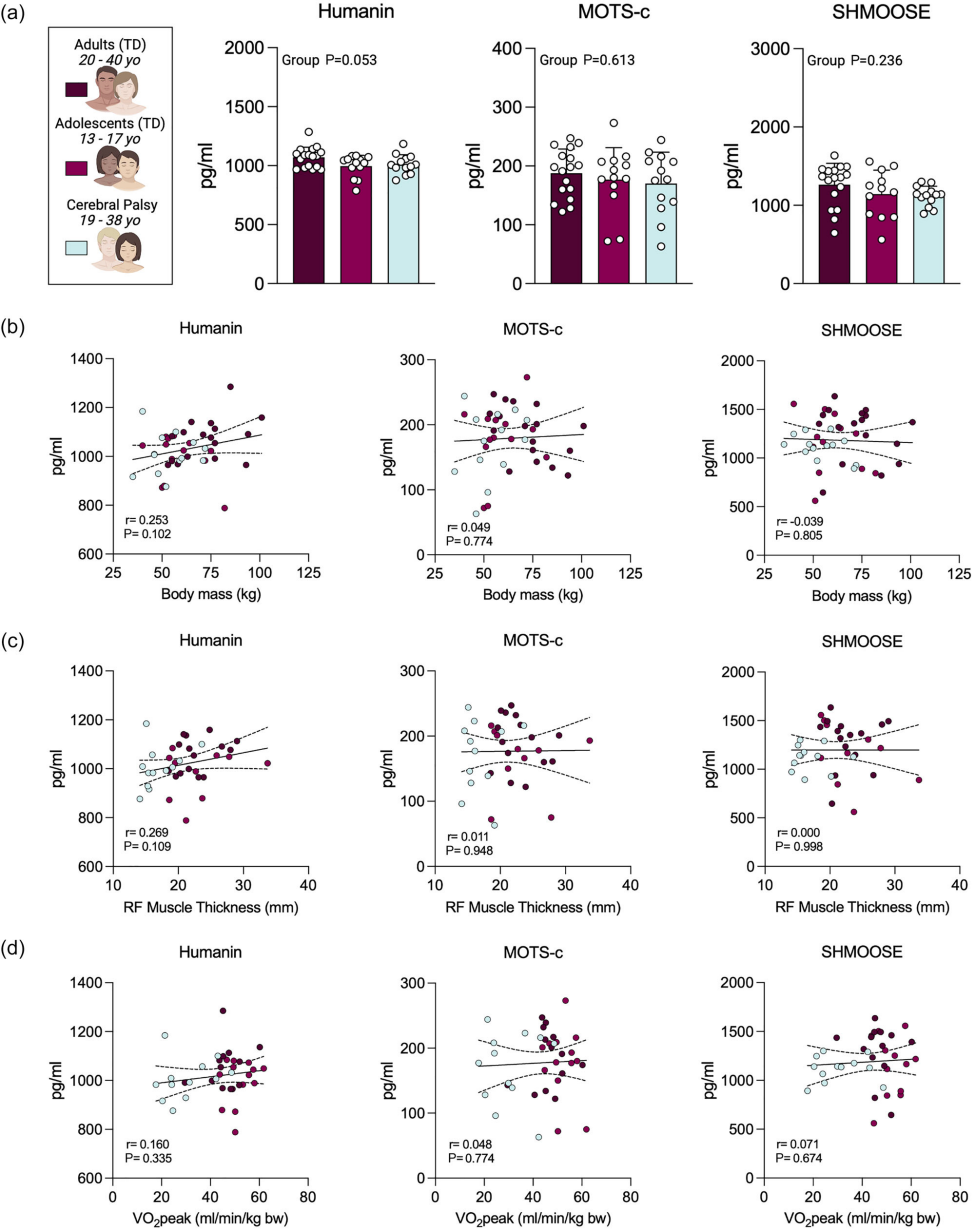
Plasma levels of mitochondrial‐derived microproteins in individuals with CP and typically developing peers. (a) Plasma levels of humanin, MOTS‐c and SHMOOSE in TD adults (*n* = 17), TD adolescents (*n* = 13) and individuals with CP (*n* = 13). (b) Plasma levels of humanin, MOTS‐c and SHMOOSE in all groups correlated with body mass. (c) Plasma levels of humanin, MOTS‐c and SHMOOSE in all groups correlated with RF thickness. (d) Plasma levels of humanin, MOTS‐c and SHMOOSE in all groups correlated with ${\dot{V}}_{O_{2}\mathrm{peak}}$. Panel (a) was analysed using a one‐way ANOVA, and panels (b–d) were analysed using Pearson's correlations. CP, cerebral palsy; MOTS‐c, mitochondrial open‐reading frame of the 12S rRNA; RF, rectus femoris; SHMOOSE, small human mitochondrial ORF over serine tRNA; TD, typically developing.

**FIGURE A1:**
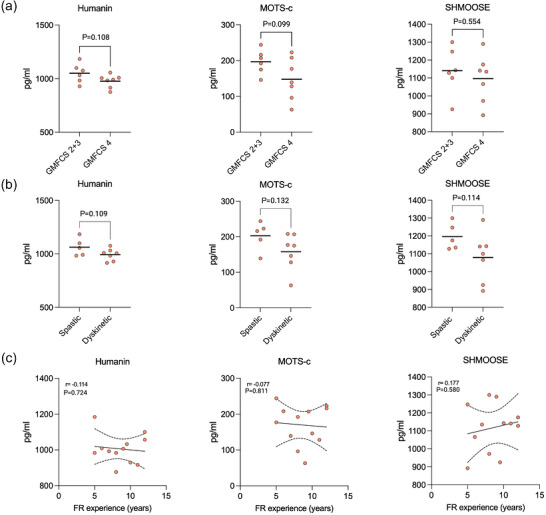
Plasma levels of mitochondrial‐derived microproteins related to clinical characteristics of CP and frame running experience. (a) Plasma levels of humanin, MOTS‐c and SHMOOSE in individuals with CP divided by GMFCS levels. (b) Plasma levels of humanin, MOTS‐c and SHMOOSE in individuals with spastic or dyskinetic CP. (c) Plasma levels of humanin, MOTS‐c and SHMOOSE in individuals with CP correlated to years of frame running experience. Panels (a, b) were analysed using an unpaired *t*‐test. Panel (c) was analysed using Pearson's correlations. CP, cerebral palsy; FR, frame running; GMFCS, Gross Motor Function Classification System; MOTS‐c, mitochondrial open‐reading frame of the 12S rRNA; SHMOOSE, small human mitochondrial ORF over serine tRNA; TD, typically developing.

Resting plasma levels of humanin (996 ± 93–1070 ± 86 pg/ml), MOTS‐c (170 ± 53–188 ± 41 pg/ml), and SHMOOSE (1116 ± 128–1263 ± 274 pg/ml) did not differ between the three groups (*P *> 0.05; Figure 2a). Levels of humanin, MOTS‐c and SHMOOSE showed no relationship to bodyweight (*r* = 0.253/0.049/0.039 for humanin/MOTS‐c/SHMOOSE, *P *> 0.05, Figure 2b), muscle thickness (*r* = 0.269/0.011/0.000, *P *> 0.05, Figure 2c), ${\dot{V}}_{O_{2}\mathrm{peak}}$ (*r* = 0.160/0.048/0.071, *P *> 0.05, Figure 2d), or reported daily activity levels (*r* = 0.227/−0.062/−0.096, *P *> 0.05, data not shown). In individuals with CP specifically, GMFCS levels (GMFCS 2+3 vs. GMFCS 4, *P *> 0.05), type of CP (spastic vs. dyskinetic, *P *> 0.05), or years of frame running experience (*r* = −0.114/−0.077/0.177 for humanin/MOTS‐c/SHMOOSE, *P *> 0.05) could not explain basal levels of the three MDPs (Appendix Figure A1).

### 3.3 Physiological responses to an acute bout of endurance exercise

Participants were asked to conduct a 45‐min endurance exercise time trial either by conventional running for TD adults and adolescents, or by frame running for adults with CP. All groups rated their perceived effort similarly across both 10−40 min of running (15 ± 2 – 16 ± 2 RPE) and the final 5 min (18 ± 1 – 19 ± 1 RPE) (Table 3). Post‐running lactate values were similar in all groups, at 5.1 ± 3.6–6.9 ± 3.0 mmol/L. The total distance covered by TD adults and adolescents was 7.6 ± 0.8–7.8 ± 1.3 km, whereas CP individuals ran a shorter distance of 4.7 ± 1.4 km (*P* < 0.05; Table 3). Likewise, individuals with CP reached a lower average HR at 82.1% compared to TD adults with 93.9% (*P *< 0.05). TD adolescents had an average HR of 87.5%, which did not differ from either TD adults or individuals with CP.

**TABLE 3:** Physiological response to the acute exercise bout across the three groups.

	Typically developed (TD)	Cerebral palsy (CP)	Typically developed (TD, <18 years)
HR at start (bpm)	95 ± 14	105 ± 27	96 ± 20
Mean HR across min 10–40 (bpm)	180 ± 7	160 ± 16^*,#^	181 ± 16
RPE at min 45	18 ± 1	19 ± 1	19 ± 1
Mean RPE across min 10–40	16 ± 1	15 ± 2	16 ± 2
Distance covered (km)	7.8 ± 1.3	4.7 ± 1.4^*,#^	7.6 ± 0.8
Lactate post (mmol/L)	7 ± 3	5 ± 4	5 ± 2
Max HR achieved (bpm)	195 ± 10	184 ± 10^*,#^	202 ± 11
% of max HR across min 10–40	93 ± 4	82 ± 6^*^	88 ± 8

Physiological response to the acute exercise bout across the three groups. Data presented as means ± SD. Data analysed using a one‐way ANOVA and Tukey's multiple comparisons test. *Significantly different from TD. ^#^Significantly different from TD (<18 years). Abbreviations: bpm, beats per minute; HR, heart rate; RPE, ratings of perceived exertion (assessed using the Borg RPE scale).

### 3.4 Plasma levels of MDPs after an acute bout of endurance exercise

**FIGURE 3:**
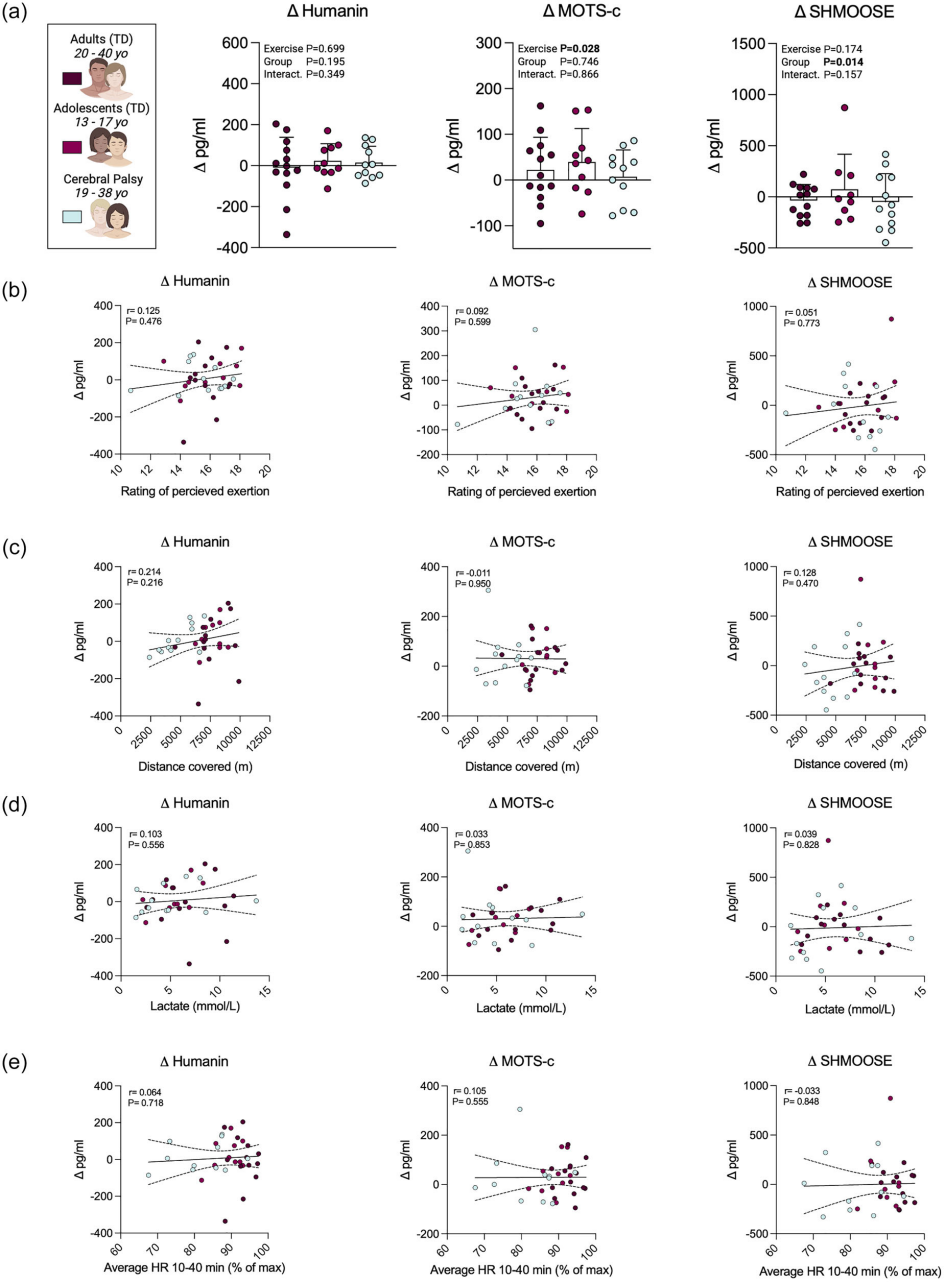
Plasma levels of mitochondrial‐derived microproteins in individuals with CP and typically developing peers after endurance exercise. (a) Delta plasma levels of humanin, MOTS‐c and SHMOOSE in TD adults, TD adolescents, and individuals with CP before and after exercise. Sample sizes were: humanin (TD adults *n* = 13, TD adolescents *n* = 10, CP *n* = 12), MOTS‐c (TD adults *n* = 13, TD adolescents *n* = 10, CP *n* = 12) and SHMOOSE (TD adults *n* = 13, TD adolescents *n* = 9, CP *n* = 12). (b) Delta plasma levels of humanin, MOTS‐c and SHMOOSE before and after exercise in all groups correlated with ratings of perceived exertion. (c) Delta plasma levels of humanin, MOTS‐c and SHMOOSE before and after exercise in all groups correlated with distance covered. (d) Delta plasma levels of humanin, MOTS‐c and SHMOOSE before and after exercise in all groups correlated with post‐exercise lactate. (e) Delta plasma levels of humanin, MOTS‐c and SHMOOSE before and after exercise in all groups correlated with average HR expressed as a percentage of max HR. Panel (a) was analysed using a two‐way repeated measures ANOVA, panels (b, e) were analysed using Spearman's rank correlation, and panels (c, d) were analysed using Pearson's correlation. CP, cerebral palsy; HR, heart rate; MOTS‐c, mitochondrial open‐reading frame of the 12S rRNA; SHMOOSE, small human mitochondrial ORF over serine tRNA; TD, typically developing.

Across all groups, plasma levels of humanin (Δ −10 ± 148–24 ± 83 pg/ml) and SHMOOSE (Δ −49 ± 277–74 ± 343 pg/ml) were unaffected by the endurance exercise (*P *> 0.05). In contrast, MOTS‐c showed a significant systemic response to physical exercise across all groups (Δ 7 ± 58–40 ± 73 pg/ml, *P *< 0.05; Figure 3a). None of the exercise intensity measures; RPE (*r* = 0.125/0.092/0.051 for humanin/MOTS‐c/SHMOOSE, *P *> 0.05, Figure 3b), distance covered (*r* = 0.214/−0.011/0.128, *P *> 0.05, Figure 3c), post‐exercise lactate (*r* = 0.103/0.033/0.039, *P *> 0.05, Figure 3d), or average HR (*r* = 0.064/0.105/−0.033, *P *> 0.05, Figure 3e) showed any association with the delta values of the three MDPs.

## 4 DISCUSSION

This study is the first to describe the levels of circulating MDPs at rest and in response to acute exercise in adults with CP. At rest, circulating concentrations of humanin, MOTS‐c and SHMOOSE did not differ from those observed in TD adults and adolescents, and showed no association with CP subtype or severity of motor impairment. Despite marked differences in physiological features previously associated with altered MDP levels, such as reduced muscle mass (Domin et al., 2023; D'Souza et al., 2020), these factors did not appear to adversely influence the basal MDP profile in the plasma of adults with CP. In this investigation, after acute exercise, circulating MDPs displayed no or modest changes across all groups. Importantly, although individuals with CP covered less distance than their TD peers, their post‐exercise MDP levels were still comparable to those of TD individuals. Collectively, these findings indicate that adults with CP display circulating MDP profiles similar to those of TD individuals, both at rest and after exercise, suggesting a preserved mitochondrial‐derived signalling capacity through MDPs.

One of the key findings of the present study was that adults with CP had basal MDP levels comparable to their TD peers, a pattern consistent for both the well‐characterised microproteins humanin and MOTS‐c and the recently identified microprotein SHMOOSE. This finding was somewhat unexpected, given the distinct physiological features of adults with CP, many of which have been implicated in shaping MDP levels (Yen et al., 2025). Skeletal muscle has been suggested as a source of MDPs (Domin et al., 2023; Lee et al., 2015), raising the possibility that the reduced muscle mass commonly observed in CP could influence circulating levels. Indeed, using rectus femoris thickness as a surrogate for muscle mass, we found ∼26% lower values in adults with CP compared with TD adults. Nonetheless, circulating concentrations of all three MDPs were comparable between groups and showed no association with muscle thickness. Although our assessments were limited to the lower extremity, these findings suggest that skeletal muscle mass is not a major determinant of endogenous plasma MDP levels. Additionally, some have suggested that aerobic capacity influences plasma MOTS‐c (Feng et al., 2025). While ${\dot{V}}_{O_{2}\mathrm{peak}}$ was ∼27% lower in adults with CP, we did not observe this affecting MOTS‐c levels, in line with previous findings from ourselves and others (Domin et al., 2023; von Walden, Fernandez‐Gonzalo et al., 2021).

In the current literature, the most consistent MDP alterations have been observed under conditions of pronounced metabolic dysregulation, such as obesity or type 2 diabetes, where reductions in circulating humanin and MOTS‐c levels are commonly reported (Du et al., 2018; Liu et al., 2019; Ramanjaneya et al., 2019; Zhou et al., 2024). Ageing may also affect MDP biology, although reported age‐related changes appear to be compartment‐specific. For example, MOTS‐c concentrations decrease in plasma but increase in skeletal muscle with ageing, whereas SHMOOSE has been reported to increase in cerebrospinal fluid (D'Souza et al., 2020; Miller et al., 2023). Although metabolic abnormalities are commonly reported in individuals with CP (Peterson et al., 2015), our cohort does not present clear evidence of such perturbations. The participants were young (mean age 25 years), maintained normal BMI (∼20 kg/m^2^), and many had extensive frame running experience (mean 7 years), with overall physical activity levels comparable to TD adults (SGPALS ∼3). These characteristics suggest a relatively low metabolic burden, which may have helped preserve basal MDP levels. Thus, the absence of group differences between CP and TD adults is consistent with evidence that metabolic or age‐related perturbations, rather than differences in muscle mass or aerobic capacity alone, are the primary drivers of shifts in MDP profiles. However, given the compartment‐specific regulation reported for several MDPs, responses in plasma may differ from those observed in other tissues, such as skeletal muscle or cerebrospinal fluid.

We did not detect any associations between circulating MDP levels and the two main CP subtypes present in this cohort (spastic and dyskinetic) or with the degree of motor impairment as classified by GMFCS. There was, however, a tendency for lower MOTS‐c concentrations in individuals at GMFCS level 4 compared with those at levels 2–3 (28% lower, *P* = 0.09), suggesting that MOTS‐c may decline with increasing motor disability. This observation is consistent with evidence that more severe motor impairment is linked to adverse blood profiles and a higher prevalence of metabolic risk factors (Heyn et al., 2019; Peterson et al., 2012). However, given the small sample size and the inherent clinical heterogeneity of CP, these findings remain preliminary and require confirmation in larger cohorts.

Despite the challenging exercise, the overall response of circulating MDPs was modest in all groups. Of the microproteins measured, only MOTS‐c showed a significant main effect of exercise, increasing by ∼17% at the 1‐h post‐exercise time point. In contrast, circulating humanin levels remained unchanged after exercise. In our previous report on TD individuals only, we found an increase in humanin, but no effect on MOTS‐c following 45 min of ergometer cycling (von Walden, Fernandez‐Gonzalo et al., 2021). This discrepancy may reflect differences in exercise mode (cycling vs. running), exercise intensity or participant characteristics, as discussed in a recent review (Atakan et al., 2024), with the eccentric component and altered muscle recruitment patterns inherent to running potentially modifying the MDP response.

Although the exercise bout induced no changes in circulating humanin and modest increases of MOTS‐c, the response observed in individuals with CP was not different from that of TD individuals. These findings suggest that, in terms of MDPs, adults with CP may retain a comparable capacity to respond to endurance exercise as their TD peers if they exercise at a comparable perceived exertion. Systemic MDP fluctuations, particularly MOTS‐c, may thus be influenced more by relative exercise intensity and perceived exertion (Borg RPE) than by absolute mechanical work performed. From a practical perspective, this supports the use of Borg RPE when prescribing and monitoring endurance exercise in individuals with CP, rather than relying solely on performance‐based metrics.

In addition to humanin and MOTS‐c, we also measured SHMOOSE, a novel microprotein recently shown to correlate with age and age‐related diseases (Miller et al., 2023). We found that basal circulating SHMOOSE levels are not altered in adults with CP. This microprotein also exhibited considerable variability in response to exercise, and our data do not support a consistent exercise‐induced response. The functional significance of this finding, however, remains unclear and requires further research.

In conclusion, despite differences in muscle mass, aerobic capacity and exercise performance, adults with CP exhibit circulating MDP levels comparable to those of TD peers, both at rest and following an acute bout of endurance exercise.

## AUTHOR CONTRIBUTIONS

Conceptualisation – Ferdinand von Walden. Participant recruitment and data collection—Linnéa Corell, Emma Hjalmarsson, Stefan M. Reitzner, Jessica Norrbom, Rodrigo Fernandez‐Gonzalo, Ferdinand von Walden. Laboratory analysis – Junxiang Wan, Pinchas Cohen. Data analysis and figures – Oscar Horwath, Sebastian Edman. Writing, original draft – Oscar Horwath, Linnéa Corell, Sebastian Edman. Writing, editing – all authors. All authors have read and approved the final version of this manuscript and agree to be accountable for all aspects of the work in ensuring that questions related to the accuracy or integrity of any part of the work are appropriately investigated and resolved. All persons designated as authors qualify for authorship, and all those who qualify for authorship are listed.

## CONFLICT OF INTEREST

None declared.

## GENERATIVE AI STATEMENT

No generative AI tools were used in the preparation of this manuscript.

## Data Availability

Data will be made available upon reasonable request.
